# Evaluation of Reducing Ability and Antioxidant Activity of Fruit Pomace Extracts by Spectrophotometric and Electrochemical Methods

**DOI:** 10.1155/2020/8869436

**Published:** 2020-12-14

**Authors:** Georgii S. Vasyliev, Victoria I. Vorobyova, Olga V. Linyucheva

**Affiliations:** Chemical Technology Department, National Technical University of Ukraine “Igor Sikorsky Kyiv Polytechnic Institute”, Kyiv 03056, Ukraine

## Abstract

The component profiles of apricot, grape, and black currant pomace extracts have been analyzed using HPLC coupled to diode-array detection and tandem mass spectrometry (HPLC-DAD-MS). The predominant components in grape, apricot, and black currant pomace extracts were phenolic acids and flavonols. The redox behavior of apricot, black currant, and grape pomace water extracts was evaluated by means of cyclic voltammetry. Also, individual substances mainly present in the extracts were analyzed. The results of electrochemical testing were compared to traditional chemical techniques of potassium ferricyanide reduction (FRAP) and phosphomolybdenum assay, and fair agreement was established. The 2,2-diphenyl-1-picrylhydrazyl (DPPH) radical scavenging assay and 2,2′-azino-bis(3-ethylbenzothiazoline-6-sulphonic acid) (ABTS) radical scavenging assays were applied in order to estimate antioxidant activity. The reducing power of the grape extract was found to be higher than that of the apricot and black currant extracts in both potassium ferricyanide reduction (FRAP) and phosphomolybdenum methods.

## 1. Introduction

Agri-food by-products produced during handling and processing of fruits and vegetables, including cakes, pomace, peels, seeds, leaves, bracts, cull, fruits, and stones, represent a major waste disposal problem for the food industry. Integrated utilization of food waste is a progressive direction of resource conservation. In almost every country in the world, the most important advances in scientific and technological progress and worldwide experience in the recycling of household and vegetable waste are used [1, 2]. Integrated use of food industry waste allows obtaining significant savings of material and energy resources, providing increased levels of closed production and resource cycles, which contributes to the economic efficiency of production. At the same time, the process of environmental pollution by the production of waste is minimized. In the area of integrated use of food industry waste, the idea is not only to introduce low-waste technologies into the production process. The involvement of wastes in chemical technology production processes as a secondary raw material makes it possible to turn it into a valuable product, followed by its widespread use for the chemical materials, pharmaceutical, and cosmetic industries. A significant amount of waste is generated in the processing of fruit and berry crops. Almost all these wastes are secondary raw materials because they contain natural organic compounds. Therefore, the priority direction for the development of green chemical technologies is the search and production of organic compounds (plant extracts) from the waste of vegetable raw materials, as well as the study of the component composition and physicochemical properties of the obtained extracts involved in the production process of waste organic compounds obtained from the waste of vegetable raw materials.

Pomace, the press residue from fruits accumulating as waste product in the food industry, contains high amounts of “green” organic substances. Phenolic compounds and flavonoids [3], organic acids, and terpenoid compounds are the most abundant secondary metabolites of the fruits pomace and have drawn increasing attention due to the possibility of their use in various fields of chemical technology. For instance, “green” compounds can be used as antioxidants, as potential health-supporting phytochemicals [4], and “eco-friendly reductants” to obtain nanoscale materials [5–7] for the inhibition of corrosion of metals in the corrosive media [8–10] and as functional ingredients in nutraceuticals and dietary supplements [11, 12].

The reducing power of the extract is a key factor in determining its potential for producing nanoscale materials from metal ions. The inhibition and antioxidant properties of fruit pomace are due to their redox activity and reducing capacity. Despite that the reducing capacity of the main components of the extract serves as the main indicator of potential reducing activity, it is worth knowing the reducing ability of the extract as a whole. Thus, reducing capacity testing has become an important tool in the search for “green” reductants for successful uses in the various fields of chemical technology. Various methods have been applied to measure the reducing activity or redox behavior of plant-based extracts [13–20]. Different techniques, including spectrophotometric determination [19], such as “reducing power assay” and “ferric reducing power” (FRAP), are employed to evaluate the reducing ability of extracts and their main compounds [20]. The reducing power assay evaluates the reducing capacity based on the reduction of Fe (III) and Mo (VI), respectively [20].

The critical discussion on the advantages/disadvantages of these methods continues to grow; however, the electrochemical approach using cyclic voltammetry technique is attracting attention because of its simplicity, sensitivity, versatility, quick screening, cost-effectiveness, as well as mechanistic information of redox reaction and the electrochemical behavior [14–17]. Electrochemical methods for redox activity determination are less frequently used in spite of their advantages over spectrophotometric methods. Some works have reported that the lower the potential of oxidation, the stronger the reducing agent in the plant extract for synthesis of nanoparticles is. However, the literature lacks data on the redox characteristics of black currant, apricot, and grape pomace extracts determined with electroanalytical techniques.

Considering the compositional profile of apricot, black currant, and grape pomaces, which have been found to be the major source of phenolic compounds and flavonoids, organic acids, and terpenoid compounds, they have high potential to be used as “green” reductants or antioxidants in chemical technology and food industry [15–20]. Therefore, the objectives of this study were to evaluate the redox characteristics of apricot, black currant, and grape pomace extracts and their main components (gallic, chlorogenic, ascorbic, and caffeic acids; quercetin; and catechin) by use of cyclic voltammetry technique, compare the results with FRAP assay, and evaluate the antiradical activity of the extracts.

## 2. Materials and Methods

Grape, black currant, and apricot extracts were obtained from the pomaces after the mechanical pressing of the fresh fruits. The extracts were analyzed to determine the composition of the main compounds and redox activity. The fruit pomaces were supplied by an agro-food company («VINNI FRUT») from the Vinnytsia city, Ukraine.

### 2.1. Extract Preparation

The fruit pomaces were mixed with distilled water in the 1 : 10 (w/v) ratio at 25°C. The mixture was placed in an ultrasound bath. The ultrasound of 27 kHz frequency and 6 W/cm^2^ intensity was applied for 2 hours. High-intensity ultrasound caused cavitation in the solution, thus intensifying the extraction process. The air was bubbled through the solution during the extraction in order to achieve the highest reducing ability of the extract, as was established previously [21]. At the end of the process, the temperature of the solution reached 40°C due to cavitation stirring of the solution. After the extraction, the solution was filtered through a paper filter and submitted for electrochemical analysis, high-performance liquid chromatography-diode array detector-tandem mass spectrometry analysis, and the determination of the radical scavenging assays. In the case of determination of the reducing power by the phosphomolybdenum method, the filtrate was concentrated with a rotary evaporator. In this case, the mass of obtained extract was 1.55 ± 0.01 g.

### 2.2. HPLC-DAD-MS Analysis

The identification and quantification of compounds in the grape, apricot, and black currant pomace extracts were performed by high performance liquid chromatography coupled to diode-array detection and tandem mass spectrometry (HPLC-DAD-MS). For HPLC-DAD–MS analysis, 1 g of extract solution was mixed with 10 mL distilled water, followed by filtration using nylon syringe filter (pore size 0.45 *μ*m). Chromatographic separation was performed on an ACE 5 C18 column (Bath V13-7473) (250 mm × 4.6 mm, 5 *μ*m particle size). The column temperature was 35°C and the flow rate was constant at 1.5 mL/min. The mobile phase was composed of 0.1% (v/v) water: formic acid (mobile phase A) and acetonitrile (mobile phase B). The elution conditions were as follows: 0–15 min, B from 8% to 30% (5 min); 22–35 min, B from 30% to 70% (10 min); and 35–40 min, B from 70% to 8%. MS spectra were recorded using an Agilent 1290 Infinity LC System.

MS analysis was as follows: MS full-scan acquisition (*m/z* 50–1000) in negative and positive ionization modes. Voltages for the skimmer and the capillary were, respectively, 4000 and 3500 V. Other MS conditions were as follows: nebulizer gas (N_2_), 50 psi; drying gas (N_2_), 10 L/min; dry temperature, 350°C. The identity of compounds was ascertained using data from DAD and MS analyses, by comparison and combination of their retention times, UV-Vis and mass spectra, and confirmed with authentic standards when available. Chromatograms were acquired at 280, 320, 360, and 560 nm.

Quantification was performed by HPLC–DAD according to an external standard method. Furthermore, the calibration curves, limits of detection (LOD), and quantification (LOQ) of the six target compounds are shown in Table 1. Linearity response and repeatability were determined by injecting the standard solutions at six different concentrations, six times for each concentration. The calibration curves were constructed using the peak area versus concentration of the standard solutions. The linearity was evaluated by the method adjustment and the correlation coefficient value (*R*^2^). The LOD and LOQ were evaluated by the residual standard deviation, with a residual range of 2–3 and 5–10, for LOD and LOQ, respectively.

### 2.3. Cyclic Voltammetry Testing

Application of cyclic voltammetry to the reduction processes investigation in different organic compounds is based on the ability of these compounds to donate electrons. In this approach, the oxidation peak potentials *E*_p.а._ in the forward anodic scan are taken into consideration in order to rank the reducing capacity of compounds; the lower the *E*_p.а._ value, the easier the oxidation and therefore the more potent the reductant is.

The electrochemical measurements were conducted in the three-electrode cell of 50 cm^3^ volume. The working electrode was a glassy carbon electrode of 2 mm in diameter. The surface of the electrode prior to testing was polished to a shine with polishing suspension, rinsed with organic solvent, and dried with filter paper. The auxiliary electrode was a platinum plate and the reference electrode was the saturated silver chloride electrode (SSCE) attached to the cell through a salt bridge. The water extract or individual compound solution was mixed with acetate buffer (pH 4) and NaClO_4_ in the following mass ratio 70 : 28 : 2 and placed into the cell. The cell was thermostated in a water bath at 25°C. In total, 3 extracts, 7 individual compounds, and 6 mixtures were tested. The concentration of individual compounds was 0.5 mM/L and in the same amount each component was added to the mixture.

The potential scan range was 0…1 V/SSCE with a scan rate of 100 mV/s. The scan started from the OCP in cathodic direction and reversed once the potential reached 1 V/SSCE. In total, 5 cycles were measured in each solution to ensure data convergence. The values of oxidation peak potentials *E*_p.а._, reduction peak potentials *E*_p.c_, and anodic and cathodic peak currents were determined from cyclic curves.

### 2.4. Determination of the Reducing Power by Phosphomolybdenum Method

The reducing power of the apricot, black currant, and grape pomace extracts was evaluated by the phosphomolybdenum method as described by Prieto et al. [19]. In this method, Mo(VI) is reduced to Mo(V) by the antioxidant agents with the subsequent formation of a green phosphate/Mo(V) complex that shows a maximal absorption at 695 nm. The volume ratio of the extract to the reagent was 1 : 10; 0.5 mL of each solution and ascorbic acid (100 *μ*g/mL) was taken for the experiment and mixed with 5 mL of reagent (0.6 M sulfuric acid, 28 mM sodium phosphate, and 4 mM ammonium molybdate). The blank solution contained 5 mL of the reagent solution and the corresponding volume of the solvent, which was applied to the sample. All the tubes were closed and incubated in a boiling water bath at 95°C for 90 minutes. After the samples cooled to room temperature, the absorbance of each solution was measured at 695 nm against the blank using a UV–Vis spectrophotometer (UV-5800PC spectrophotometer, FRU, China). The reducing capacity is expressed as the number of equivalents of ascorbic acid per gram of dry extract (mg AAs eq/dry extract).

### 2.5. Determination of Reducing Power Based on the Reduction of Fe (III)

Fe (III) reduction is often used as an indicator of the electron donating activity, which is an important indicator of the phenolic-reducing capacity [20]. Extracts that have reduction potential react with potassium ferricyanide (Fe^3+^) to form potassium ferrocyanide (Fe^2+^), which then reacts with ferric chloride to form a ferric ferrous complex that has an absorption maximum at 700 nm. To prepare the reaction solution, different amounts of the extract (0.005, 0.01, 0.015, 0.02, and 0.025 g) were dissolved in an appropriate solvent (methanol 1 mL) plus 1 mL phosphate buffer (0.2 M, pH 6.6) and 1 mL of potassium ferricyanide solution (1%). The resulting solution was incubated at 50°C for 20 minutes. Then, 1 mL of trichloroacetic acid (10%) was added to terminate the reaction and the tube was cooled under running water for 5 min. The resulting mixture was centrifuged at 3000 rpm for 10 min. An aliquot of 2 mL was then removed from the top layer of each solution, to which 2 mL of distilled water and 0.4 mL of ferric chloride solution (0.1%) were added. The solution absorbance was measured at 700 nm. Increasing the absorbance of the reaction mixture indicates an increase in the reducing power.

### 2.6. 2, 2-Diphenyl-1-picrylhydrazyl (DPPH) Radical Scavenging Assay

The free radical scavenging activity was determined by the capacity of the extracted polyphenols to reduce the stable free radical 2, 2-Diphenyl-1-picrylhydrazyl (DPPH) [14]. The extract was dissolved in ethanol (EtOH) at various concentrations. Each dilution (0.5 mL) was mixed with 3 mL of EtOH solution of DPPH (0.1 mmoL). The mixture was incubated in the dark at room temperature. The absorbance of the DPPH solution was measured at *λ* = 517 nm (*A*_control_) and 30 minutes after adding the extract (*A*_sample_). In the blank, ethanol was used in place of the sample; ВНТ (butylated hydroxyl toluene) was used as a positive control. The ability to scavenge the DPPH-free radical was calculated using the following equation:

(1) $$\begin{array}{c} \text{DPPH radical scavenging activity}\left(\text{\%}\right)=\frac{A_{\text{control}}-A_{\text{sample}}}{A_{\text{control}}}, \end{array}$$

where *A*_control_ is the absorbance of DPPH radical + ethanol and *A*_sample_ is the absorbance of DPPH radical with extract/standard.

### 2.7. 2,2′-Azino-bis(3-ethylbenzothiazoline-6-sulphonic Acid) (ABTS) Radical Scavenging Assay

Scavenging of 2,2′‐Azino‐bis‐(3‐ethylbenzothia‐zoline‐6‐sulf‐onic acid) (ABTS) radicals was performed according to the methodology described in Thaipong et al. [20]. A stock solution was prepared by adding 10 mL of 7.4 mM ABTS solution to 10 mL of 2.6 mM K_2_S_2_O_8_ and left at room temperature in the dark for 15 h and then stored at −20°C until required. The working solution was freshly prepared by diluting 1 mL of stock solution with approximately 60 mL of ethanol to obtain an absorbance value of 1.1 ± 0.02 at 734 nm on the day of analysis. The extract was dissolved in ethanol at various concentrations ranging from 0.01 to 0.5 mg/mL. Each dilution (0.1 mL) was mixed with 3 mL of reagent and the absorbance of the ABTS cationic radical was measured at *λ* = 734 nm (*A*_control_) and 60 minutes after adding the extract (*А*_sample_). Trolox was used as a positive control (*y* = 37.284*x* (*R*^2^ = 0.9997). The scavenging activity was calculated using the following equation:

(2) $$\begin{array}{c} \text{ABTS radical scavenging activity}\left(\%\right)=\left(A_{\text{control}}-\frac{A_{\text{sample}}}{A_{\text{control}}}\right)n100. \end{array}$$

### 2.8. Statistical Analysis

Averaged result of three replicates for each sample was used for the subsequent statistical analysis. The linear regression model was proposed using the software Matlab.

## 3. Results and Discussion

**Figure 1:**
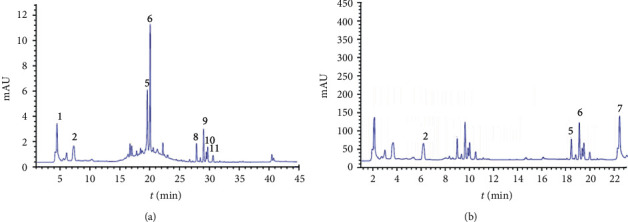
HPLC-DAD profile (280 nm (a) and 320 (b) nm) of the apricot pomace extract.

**Figure 2:**
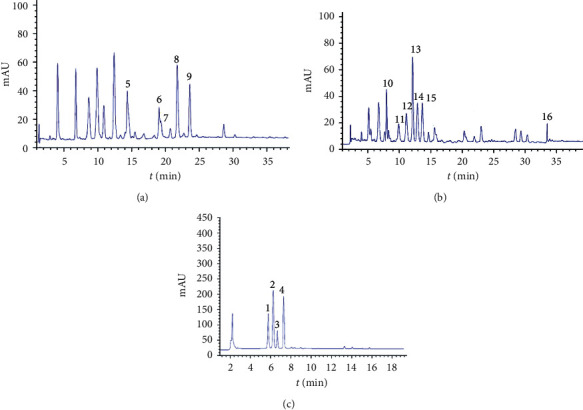
HPLC-DAD profile 320 nm (a), 360 nm (b), and 560 nm (c) of the black currant pomace water extracts.

**Figure 3:**
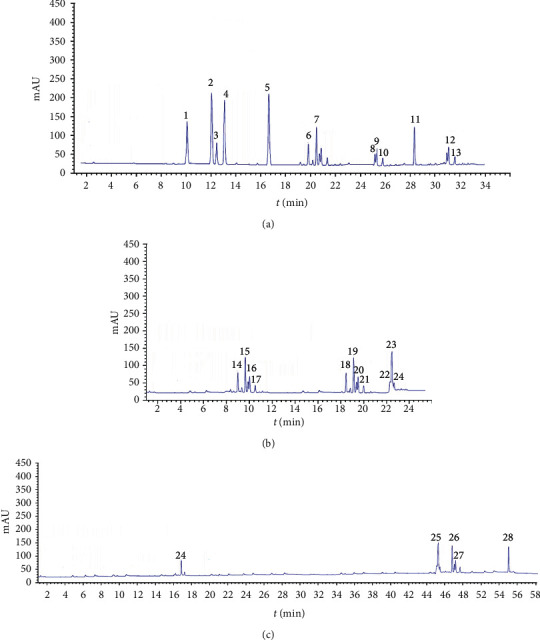
HPLC-DAD profile (560 nm (a) and 280 (b) nm 370) of the grape pomace extract.

The corresponding chromatograms obtained for the grape, apricot, and black currant pomace extracts at several wavelengths are presented in Figures 1–3. A list of the detected compounds, retention times, mass spectra, and *λ* max (UV-Vis) are presented in Tables 2–4.  Figure 1 shows a typical chromatographic profile of apricot pomace extract demonstrating the separation of multiple phenolic and flavonol components. The main components of the apricot pomace extract were hydroxycinnamic acids: peaks 2, 4, 5, 6, and flavonols: peaks 7 and 9 (Table 2). A significant amount of anthocyanin glycosides were identified in apricot pomace extract: myricetin and myricetin 3-O-glucoside. The compounds that had a [M-H]- at *m/z* 447 and *λ* max 346 nm were identified as kaempferol 3-O-glucoside. The peak with [M-H]^−^ in the negative mode at *m/z* and MS^2^ fragment ion at 331 was identified as a quinic acid. (+)-Catechin with deprotonated molecule [M-H]^−^ at *m/z* 289 and MS^2^ ions at *m/z* 245, 205 was detected with *λ* max 244 and 276 nm. Compound 4 with deprotonated molecular ion at *m/z* 353 and base peak at *m/z* 191 was characterized as 3-quinic acid. Compounds 5 and 6 (neochlorogenic acid and chlorogenic acid) were identified by comparison with analytical standards.

The preliminary LC-MS study of black currant pomace water extract identified anthocyanins, flavonols, and flavan-3-ols [21]. Thus, the absorbance changes were monitored at 320 nm, 360 nm, and 560 nm (Figure 2, Table 3). Significant amounts of hydroxycinnamic acids (caffeic (peak 5), chlorogenic (peak 10), and neochlorogenic acids (peak 9) were identified.

The most abundant anthocyanin in black currant pomace water extract was delphinidin, with two derivatives (Delphinidin-3-O-glucoside (peak 1), Delphinidin-3-O-rutinoside (peak 3)). These results were in accordance with published data for detected anthocyanins in black currant pomace water extracts [21].

Figure 3 presents an HPLC chromatogram (DAD: 280, 320, and 560 nm) showing the profiles of the main compounds obtained from a grape pomace extract. Qualitative and quantitative analyses of the extract by HPLC-DAD identified the following phenolic acids: caffeic, *p-*coumaric, sinapic, and ferulic. Also, the highest content of anthocyanins (malvidin 3-O-glucoside, quercetin-3-O-glucoside, and quercetin-3-O-rutinoside), anthoxanthins, and stilbenes was observed. (+)-Catechin was identified in all extract of pomaces (apricot, black currant, and grape). Quercetin derivatives were dominant flavonols in grape extract (Table 4). At a retention time of 55.7 with *λ* max 301 and 151 nm, quercetin having [M + H]^+^ of *m/z* 301 was identified from the conformation of MS^2^ fragmentation by splitting the parental molecule into *m/z* 301 and *m/z* 151. Three free hydroxycinnamic acids were identified in all the extracts: caffeic acid, chlorogenic (3-O-caffeoylquinic acid), and neochlorogenic acid (5-O-caffeoylquinic acid). Caffeic acid was eluted at retention time of 4.03 min with 325 nm and identified with *m/z* 181.

The analytical standards were unavailable for all separated peaks/compounds.  Table 5 shows the content of selected compounds: quercetin, сaffeic acid, сhlorogenic acid, gallic acid, and (+)-catechin, identified and quantified by HPLC-DAD-MS. The black currant pomace extract is characterized by its abundance of caffeic acid and chlorogenic acid. In the case of the extract from grape pomace, (+)-сatechin and сhlorogenic acid were the dominating constituents; this content amounted to around 479.7 *μ*g/g and 604.51 *μ*g/g. The grape pomace presented the highest levels of gallic acid (1025.0 *μ*g/g). As shown in Table 4, gallic acid (804.4 *μ*g/g) was the major polyphenol present in the apricot pomace extract followed by chlorogenic acid (704.65 *μ*g/g), (+)-catechin (254.1 *μ*g/g), and caffeic acid (108.2 *μ*g/g).

**Figure 4:**
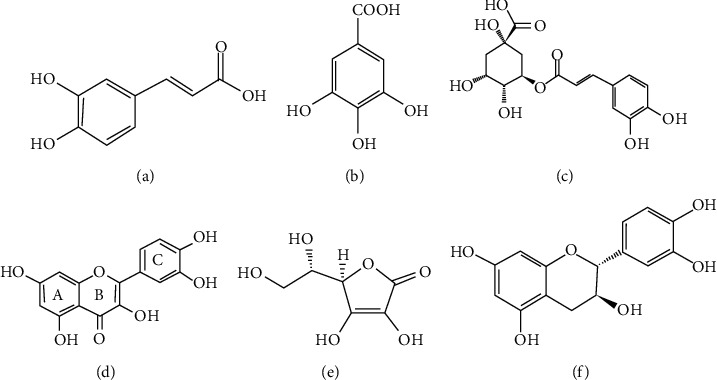
Chemical structures of the main chemical constituents of apricot, black currant, and grape pomace extracts. (a) Caffeic acid. (b) Gallic acid. (c) Chlorogenic acid. (d) Quercetin. (e) Ascorbic acid. (f) (+)-Catechin.

Thus, the apricot, black currant, and grape pomace extracts mainly consist of phenolic and flavonol components (Figure 4). These results clearly indicate that the phenolic acids and flavonol derivatives are the main compounds in the pomace extracts, which is in agreement with the findings of previous studies [22–26].

### 3.1. Voltammetric Behavior of the Apricot, Black Currant, and Grape Pomace Extracts

**Figure 5:**
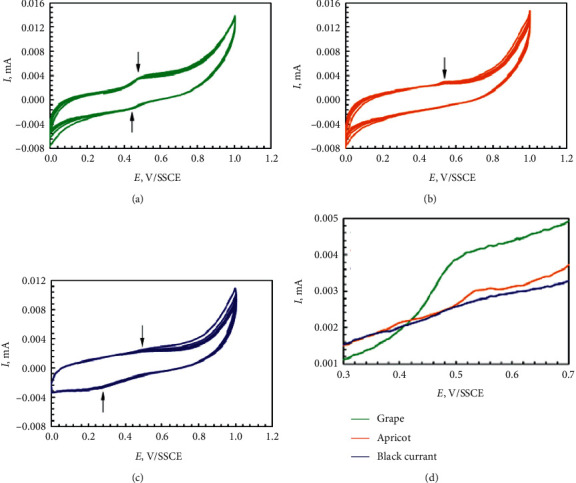
Cyclic voltammograms for the grape (a), apricot (b), and black currant (c) pomace extracts.

The electrochemical oxidation of the apricot, black currant, and grape pomace extracts and their main components such as gallic, chlorogenic, caffeic, and ascorbic acids; quercetin; and catechin was performed. The cyclic voltammograms for the apricot, black currant, and grape pomace extracts are given in Figure 5. The voltammograms for all the extracts showed no clear peaks but only a monotonous increase of current in the potential range 0.6 – 1.0 V/SSCE. Both black current and grape pomace extracts give quasi-reversible shoulders; however, apricot extract gives only an anodic one. Similar results were obtained in our previous study [15]. Several parameters can be extracted from the cyclic voltammetry curves to characterize the reducing ability of the extracts (Table 6).

The top scan represents the oxidation of the compounds that are contained in the extracts, generating a positive (anodic) current *I*_p.a._ = 2.3–3.5 *μ*A, peaking electrode potentials of *E*_p.a.1_ = 0.54 V/SSCE, *E*_p.a.1_ = 0.51 V/SSCE, and *E*_p.a.1_ = 0.48 V/SSCE for black currant, apricot, and grape pomace extracts, respectively (Table 6). On the reverse scan, a negative (cathodic) peak is reproduced for black currant and grape pomace extracts at *E*_p.c.1_ = 0.28 V/SSCE and *E*_p.c.1_ = 0.44 V/SSCE. The absence of a cathodic peak in the reverse scan for apricot pomace extract gives information about the nonreversibility of the redox reaction of the oxidized compound generated in the forward scan [27]. Thus, the reducing capacity of tested extracts decreases as follows: grape pomace extract > apricot pomace extract > black currant pomace extract.

**Figure 6:**
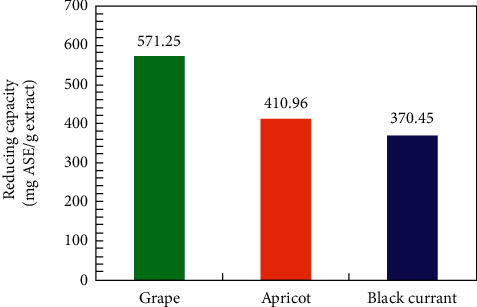
Reducing power of the aqueous black currant, apricot, and grape pomace extracts evaluated by the phosphomolybdenum method.

Similar findings were also observed with the phosphomolybdenum method, where the samples of apricot and grape pomace extracts were more active.  Figure 6 shows that the grape pomace extract has the highest reducing activity (571.25 mg of ascorbic acid equivalent per gram of dry extract) compared to the other extracts that showed antioxidant capacity in the order: apricot pomace (410.96 mg of ascorbic acid equivalent per gram of dry extract) and black currant (370.45 mg ascorbic acid equivalent per gram of dry extract).

**Figure 7:**
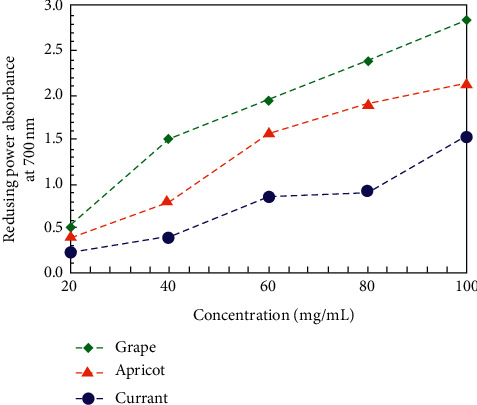
Reducing power of the aqueous black currant, apricot, and grape pomace extracts evaluated by the FRAP assay.

The reducing power assay is often used to evaluate the ability of the compounds to reduce Fe^3+^ to Fe^2+^.^.^The results demonstrated that all the extracts possessed the ability to reduce Fe_3+_ to Fe_2+_ by electron transfer reaction.  Figure 7 shows the reducing power of the extracts as a function of their concentration. The reducing power of the extracts increased with concentration. In all cases, the black currant pomace extract showed the lowest FRAP value of reducing power.

**Figure 8:**
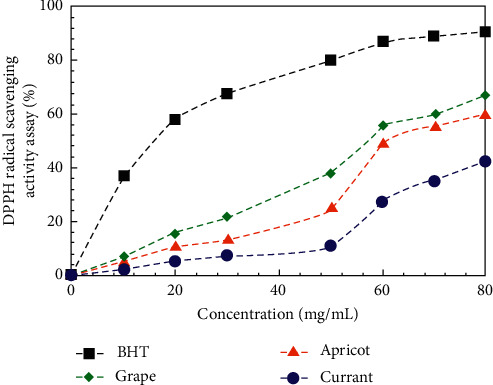
DPPH radical scavenging activity of the aqueous black currant, apricot, and grape pomace extracts.

**Figure 9:**
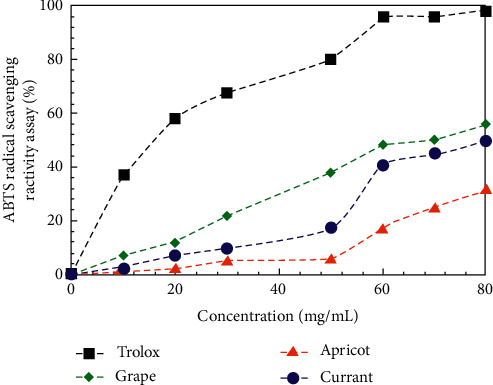
ABTS radical scavenging activity of the aqueous black currant, apricot, and grape pomace extracts.

The reducing power is often used as an indicator of electron donating activity, which is an important approach for testing the antioxidative action/antioxidant properties/radical scavenging ability of the extracts. To compare the antioxidant potential of the aqueous black currant, apricot, and grape pomace extracts, 2,2-diphenyl-1-picrylhydrazyl (DPPH) radical scavenging assay and 2,2′-azino-bis(3-ethylbenzothiazoline-6-sulphonic acid) (ABTS) radical scavenging activities were tested and the results are presented in Figures 8 and 9.

ВНТ (butylated hydroxyl toluene) and Trolox (6-hydroxy-2,5,7,8-tetramethylchroman-2-carboxylic acid) were used as positive controls. Also, 2,2-diphenyl-1-picrylhydrazyl radical scavenging assay and 2,2′-azino-bis(3-ethylbenzothiazoline-6-sulphonic acid) scavenging activity of all extracts was concentration-dependent. From the analysis of Figures 8 and 9, it can be concluded that nonlinear (S-shaped) relationship between the concentration of the extract and the DPPH and ABTS radical scavenging activity was observed. S-shaped course of curves can be explained by the complicated mechanism of the reaction. The antioxidant activity of various compounds depends on the concentration that is attributed to the structural features of the molecules [14, 28–38]. The number of active groups in a single molecule, participating in radical scavenging, increases with the increase of the concentration of this substance.

The results showed that the grape pomace extract has a strong ability to scavenge DPPH and ABTS radicals. It was found that the percentage of scavenging activity of the DPPH radicals increases in order: black currant < apricot < grape pomace extracts. At the same time, the ABTS radicals scavenging activity was found to increase in order: apricot < black currant < grape pomace extracts. The DPPH scavenging activity (%) of the grape pomace extract was 67.4% at 80 mg/mL. In the present study, ABTS scavenging activity (%) of extracts was found to vary between 31.5 and – 55.7% at a concentration of 80 mg/mL (Figure 9). Furthermore, the highest value was observed in the DPPH activity, indicating that extracts are more efficient to scavenge DPPH radicals than to scavenge ABTS radicals. These results suggest that the radical scavenging ability of the extracts is selective about the different radicals.

The values of reducing power and radical scavenging activity of the extract determined with different techniques are compared in Table 7.

Both electrochemical and spectrophotometric techniques showed the same order of reducing power of the extracts (grape pomace > apricot pomace > currant pomace). The obtained results demonstrate a correlation between the reducing power of the aqueous black currant, apricot, and grape pomace extracts evaluated by the phosphomolybdenum method; FRAP assay and antioxidant potential evaluated by DPPH and ABTS assays; and similarity of results with electrochemical methods. This points out that the electrochemical approach can be utilized as a tool for redox characterization of the extracts. The fact that an antioxidant is a reductant in its interaction with radicals generally means that it can be oxidized on the electrode surface. It has been found that extracts with strong scavenging capacities are oxidized at relatively low potentials.

### 3.2. Voltammetric Behavior of the Individual Compounds

**Figure 10:**
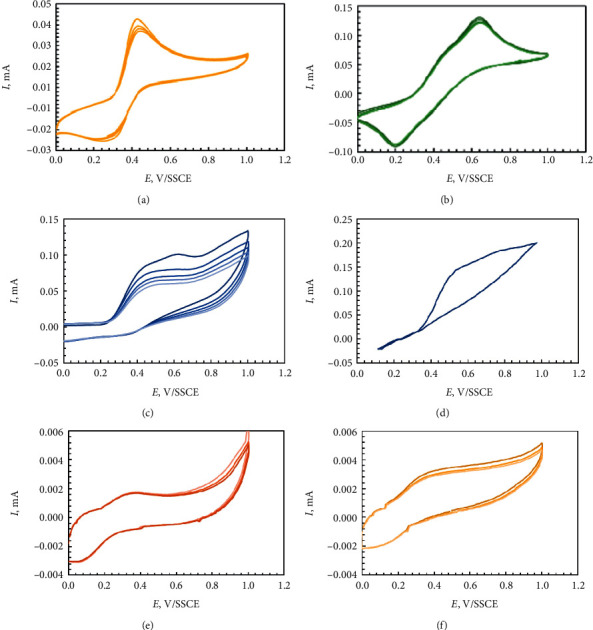
Cyclic voltammograms of 0.5 mM/L caffeic acid (a); 0.5 mM/L chlorogenic acid (b); 0.5 mM/L quercetin (с); 0.5 mM/L gallic acid (d); 0.5 mM/L(+)-catechin (e); and 0.5 mM/L ascorbic acid (f) at scan rate of 100 mVs^−1^ in acetate buffer 0.1 M (pH 4) and NaClO_4_ (70 : 28 : 2). Scan rate – 100 mVs^−1^.

Cyclic voltammograms of the individual compounds are given in Figure 10. The cyclic voltammograms present well-defined anodic peaks and a reverse peak is observed in the case of caffeic acid, ascorbic acid, сhlorogenic acid, and catechin. The ascorbic and gallic acids have only one oxidation peak.

The cyclic voltammograms for the quercetin and gallic acids showed an irreversible anodic peak of oxidation at +0.58 V and +0.60 V, respectively. On the reverse scan, no cathodic peaks were observed. It was found the *E*_p.a.1_ of the studied compounds increases in the order: caffeic acid < quercetin < сhlorogenic acid < gallic acid.

**Scheme 1:**
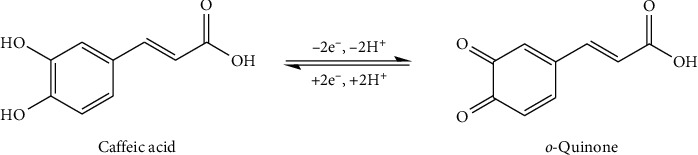
Electrochemical oxidation of caffeic acid to *o*-quinone [28].

Cyclic voltammograms of phenolic acids are displayed in Figures 10(a) and 10(b). It can be noted that phenolic acids have different electrochemical characteristics, which can be attributed to their different molecular structures [28]. The voltammograms obtained for caffeic acid present the quasi-reversible redox reaction and showed cathodic peak at *E*_p.c.1_ = 0.27 V/SSCE, with a corresponding anodic peak at *E*_p.a.1_ = 0.43 V/SSCE. The clear oxidation peak and a corresponding reduction peak can be explained by the oxidation of *o*-hydroquinone derivatives to *o*-quinone derivatives as shown in Scheme 1. It is generally known that caffeic acid is oxidized to quinone via semiquinone form. These results agree with relevant literature [28].

The cyclic voltammogram obtained for chlorogenic acid exhibits a quasi-reversible redox reaction and showed two cathodic peaks at *E*_p.c.1_ = 0.37 and *E*_p.c.2_ = 0.21 V/SSCE that corresponded to two anodic peaks at 0.47 and 0.65 V/SSCE. On the reverse scan, a negative (cathodic) peak is observed when the oxidized form of the phenolic compounds is reduced back to its original form. The chlorogenic acid has a higher potential of oxidation than the gallic acid.

**Scheme 2:**
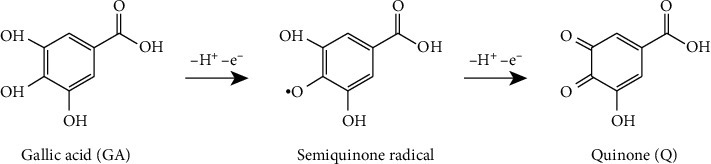
Electrochemical oxidation of gallic acid [24].

The voltammograms obtained for gallic acid (Figure 10(d)) present only one irreversible wave of oxidation at +0.52 V/SSCE. The oxidation proceeds irreversibly, as evidenced by the absence of steps on the cathodic branches of the voltammograms (Figure 10(d)). The oxidation peak is attributed to the formation of semiquinone radical (Scheme 2) [24].

The quercetin oxidation processes proceed in a cascade mechanism and is related with the catechol groups and the three hydroxyl groups in the structure of the compound [30, 31]. The cyclic voltammogram obtained for quercetin (Figure 10(c)) showed two characteristic peaks in the anodic wave: the first at 0.47 V/SSCE and the second one at 0.62 V/SSCE. These peaks are related to the oxidation of the hydroxyl group in the rings A and C. Cyclic voltammogram of quercetin presents only one cathodic peak at *E*_p.c.1_ = 0.38 V/SSCE.

**Scheme 3:**
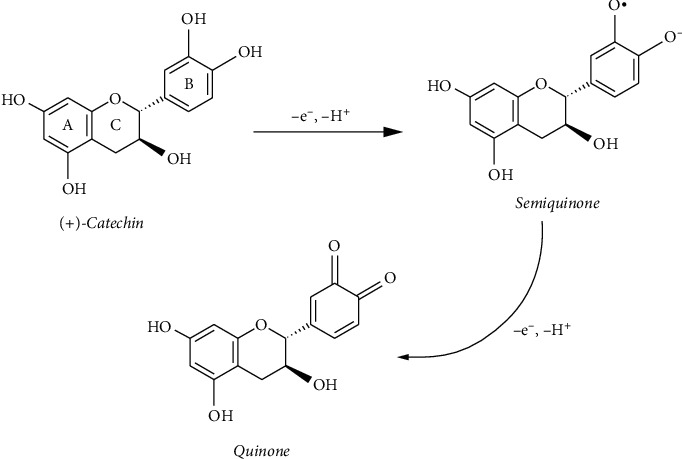
Electrochemical oxidation of catechin [34–36].

The terpenoid compounds showed the same electrochemical behavior and contained one quasi-reversible wave of oxidation at 0.39 V/SSCE and reduction at 0.16 V/SSCE. These results agree with relevant literature [32–36]. The cyclic voltammogram obtained for catechin (Figure 10(e)) exhibits a quasi-reversible redox reaction and showed that one anodic peak at 0.37 V/SSCE, which is associated with the oxidation of the B-ring of *o*-phenolic groups (Scheme 3), and the cathodic peak (reduction peak) at Ep.c.1 = 0.08 V/SSCE, which corresponds to the reduction of the oxidation products, are formed in the oxidation process (peak 1). The voltammograms obtained for L-ascorbic acid (AA) (Figure 10(g)) present the peak at 0.37 V/SSCE and is attributed to ascorbic acid oxidation.

The oxidation potentials of the apricot, black currant, and grape pomace extracts were compared with the oxidation potentials of some of their main components such as gallic, chlorogenic, and caffeic acids; quercetin; and their mixtures (Table 2). The anodic peaks of oxidation on the cyclic voltammograms of the extracts are located close to the oxidation potentials of the individual compounds, which confirm that all extracts contain these compounds. The same compounds are oxidized in the apricot, black currant, and grape pomace extracts, representing total reducing activity.

Cyclic voltammetry has been used to evaluate redox reaction and the electrochemical behavior of several polyphenol mixtures (caffeic acid and (+)-catechin) and nonpolyphenolic compounds (cysteine and L-ascorbic acid) [37]. The antioxidant properties of some binary mixtures (gallic acid/caffeic acid; gallic acid/chlorogenic acid; gallic acid/quercetin; caffeic acid/quercetin; caffeic acid/chlorogenic acid; chlorogenic acid/quercetin; (–)-catechin/caffeic acid caffeic acid/quercetin) have been studied.

**Figure 11:**
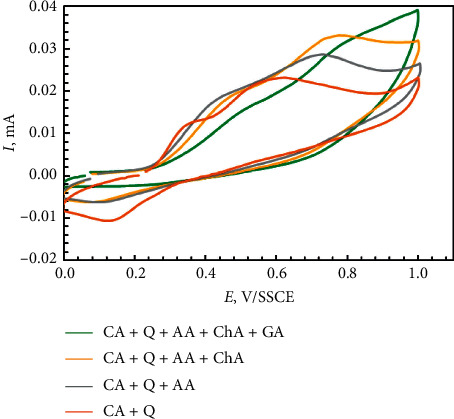
Cyclic voltammograms of a mixture of phenolic acids and monoterpenoid phenols at 0.5 mM/L concentration in acetate buffer 0.1 M (pH 4) and NaClO_4_ (70 : 28 : 2). CA, caffeic acid; Q, quercetin; AA, ascorbic acid; ChA, chlorogenic acid; GA, gallic acid. Scan rate – 100 mVs^−1^.

It is advisable to study a more complex system, which includes phenolic acids and monoterpenoid phenols. Concerning the appearance of voltammograms obtained for mixtures, some differences were observed compared to individual compounds. Voltammograms for the mixtures are shown in Figure 11.

The oxidation potentials of the mixtures are given in the increasing order (Table 8). It was observed that most individual compounds and mixtures thereof were oxidized at close potentials of about 0.4–0.6 V/SSCE. Therefore, it is natural that the cyclic voltammograms of the extracts do not have pronounced peaks, and a monotonic increase in the current density is observed, which corresponds to the total value of the individual components. Quercetin has two anodic peaks, so the mixture of Quercetin with Caffeic acid showed two anodic peaks, while Caffeic acid alone has only one anodic peak. Addition of Ascorbic acid increased the peaks' current but does not change the course of the curve. Сhlorogenic and Gallic acids have two oxidation peaks as individual compounds, so the mixture of Caffeic acid (CA) + Quercetin (*Q*) + L-ascorbic acid (AA) + Chlorogenic acid (ChA) exhibits two peaks, one with an *E*_p.a.1_ of 0.47 V and another at *E*_p.c.2_ of 0.76 V. The *E*_p.a.1_ value of the mixture is the same as for Chlorogenic acid, while the *E*_p.c.2_ is slightly different (*E*_p.c.2_ = 0.65 V/SSCE). Gallic acid, added to the mixture, increased the reducing power and this correlates with the cyclic voltammogram for the pomace extracts that have a similar monotonic course of curves. This observation shows that there is no interaction between the oxidation processes when the compounds are present in a mixture. Alternate mixing of components and the formation of a multicomponent mixture did not show any effect of pronounced synergy.

## 4. Conclusions

The major components in grape, apricot, and black currant pomace extracts were phenolic acid (caffeic acid (108.2–624.1 *μ*g/g), chlorogenic acid (517.02–704.65 *μ*g/g), gallic acid (804.4–1025 *μ*g/g), flavonols ((+)-catechin (254.1–479.7 *μ*g/g), and quercetin (1.87–2.18 *μ*g/g)).

The reducing activities of the extracts were measured by the FRAP and phosphomolybdenum methods and their oxidation potentials were determined with cyclic voltammetry. The grape, apricot, and black currant pomace extracts showed high reducing ability (370.45–571.25 mg of ascorbic acid equivalent per gram of dry extract). The results showed significant similarity of reducing power between electrochemical and spectrophotometric techniques for plant extract, which indicates that the voltammetry technique can be utilized as a simple and reliable approach to evaluate the reducing activity.

The voltammetric profile of the extracts was similar to the one obtained for each individual compound. Catechin and ascorbic acid have the lowest oxidation potential (0.37 V/SSCE) and can be named the most powerful “green” reductants. The voltammograms for the extracts showed a broad anodic peak with similar (anodic) current *I*_p.a._ = 3 *μ*A for all apricot, black currant, and grape pomace extracts. The results demonstrate that the grape, apricot, and black currant pomace extracts were found to be effective scavengers of 2,2-diphenyl-1-picrylhydrazyl (DPPH) and 2,2′-azino-bis(3 ethylbenzothiazoline-6-sulphonic acid) radicals. The mixture of individual compound shows the most reducing tendency among all the tested compounds and their mixtures.

The results of this study provide evidence that apricot, black currant, and grape pomace extracts possess reducing power. According to the results of the present study, both ferric reducing power and phosphomolybdenum methods showed that the water extract of grape pomace appeared to have the highest reducing power than that of the apricot and black currant pomaces. The reducing power decreased in the following order: grape pomace > apricot pomace > black currant pomace.

## Figures and Tables

**Table 1:** Analytical characteristics of the calibration graphs.

Compound	Calibration curve		Method LOD (*μ*g/g)	Method LOQ (*μ*g/g)
	Linear range (*μ*g/mL)	Correlation coefficient		
Caffeic acid	*y* = 11.04*x* + 14.43	0.9997	3.15	10.50
Chlorogenic acid	*y* = 15.01*x* + 22.24	0.9995	0.55	1.82
Gallic acid	*y* = 3.72*x* − 25.46	0.9988	1.46	4.88
(+)-Catechin	*y* = 1.45*x* + 1.95	0.9974	3.96	13.20
Quercetin	*y* = 14.68*x* − 1 8.25	0.9997	1.23	4.10

**Table 2:** Characterization of the main compounds of the apricot pomace extract using their spectral characteristic in HPLC-DAD-MS.

Peak	Rt/min	Mode of ionization [M+H]^+^/[M−H]^−^	Fragments MS^2^	UV-Vis max	Compound
*Phenolic acids*					
1	4.03	—/169	125, 107, 97, 79	280	Gallic acid^*∗*^
2	6.08	—181	135, 163	332	Caffeic acid^*∗*^
3	7.02	—/153	158; 189	220	1-Caffeoylquinic acid
4	7.10	—/353	191	280	Quinic acid^*∗*^
5	19.1	—/353	191	320	Neochlorogenic acid^*∗*^
6	20.2	—/353	191	244, 324	Chlorogenic acid^*∗*^

*Flavonols*					
7	22.8	—/447	284, 255, 227	265, 346	Kaempferol 3-O-glucoside
8	26.5	—/479	316, 271	258, 357	Myricetin 3-O-glucoside
9	27.8	—/317	317, 151	253, 372	Myricetin
10	28.5	—/289	245, 205	244, 276	(+)-Catechin^*∗*^
11	31.8	—/609.0	300.8, 299.9	280	Rutin

**Table 3:** Characterization of the main compounds of the black currant pomace extract using their spectral characteristic in HPLC-DAD.

Peak	Rt/min	Mode of ionization [M+H]^+^/[M−H]^−^	Fragments MS^2^	UV-Vis max	Compound
*Anthocyanins*					
1	5.84	465/—	303	277, 526	Delphinidin-3-O-glucoside
2	6.02	595/—	449, 287	280, 520	Cyanidin-3-O-glucoside
3	6.41	611/—	276, 528	465, 303	Delphinidin-3-O-rutinoside
4	7.51	655/—	311	278, 528	Malvidin 3-O-glucoside

*Phenolic acids*					
5	14.89	—/181	135, 163	332	Сaffeic acid^*∗*^
6	18.45	—/163	119	230, 310	*p-*Coumaric acid
7	19.22	—/353	191	280	Ferulic acid
8	24.32	—/353	191	320	Neochlorogenic acid^*∗*^
9	29.10	—/353	191	244, 324	Chlorogenic acid^*∗*^

*Flavonols*					
10	8.55	—/479	316, 271	258, 357	Myricetin-3-O-glucoside
11	10.02	—/463	300, 255, 151	256, 354	Quercetin-3-O-glucoside
12	11.47	—/609	300	254, 353	Quercetin-3-O-rutinoside
13	12.38	—/317	317, 151	253, 372	Myricetin
14	28.5	—/289	245, 205	244, 276	(+)-Catechin^*∗*^
15	14.62	—/447	284, 255, 227	265, 346	Kaempferol-3-O-glucoside
16	33.11	—/301	301, 151	254, 371	Quercetin^*∗*^

^*∗*^Verified with pure standard. Rt: retention time.

**Table 4:** Characterization of the main compounds of the grape pomace extract using their spectral characteristic in HPLC-DAD-MS.

Peak	Rt/min	Mode of ionization [M+H]^+^/[M−H]^−^	Fragments MS^2^	UV-Vis max	Compound
*Anthocyanins*					
1	10.01	465/—	303	277, 526	Delphinidin 3-O-glucoside
2	12.25	595/—	449, 287	280, 520	Cyanidin-3-O-glucoside
5	16.98	—/493	311	278, 528	Malvidin 3-O-glucoside
6	20.10	479/−	317	278, 527	Petunidin 3-O-glucoside
7	21.42	—	—	—	Unknown
8	24.87	—	—	—	Unknown
9	25.32	—	—	—	Unknown
10	26.91	—	—	—	Unknown
11	29.53	—	—	—	Unknown
12	31.53	—/609	301	520	Peonidin 3-O-*p*-coumaroylglucoside
13	32.54				Unknown

*Phenolic acids*					
14	5.8	—/169	125, 107, 97, 79	280	Gallic acid^*∗*^
15	12.7	—/153	109	280	Protocatechuic acid
16	18.0	—/137	93	280	*p-*Hydroxybenzoic acid
17	20.8	—/295	163	280	Coutaric acid
18	23.4	—/179	135	280	Caffeic acid^*∗*^
19	25.4	—/289	245	280	Epicatechin
20	25.7	—/197	153/182	280	Syringic acid
21	31.0	—/163	119	230, 310	*p*-Coumaric acid^*∗*^
22	31.0	—/223	164/208	280	Sinapic acid
23	32.5	—/193	134	280	Ferulic acid

*Flavanols*					
24	17.5	—/289	245	244, 276	(+)-Catechin^*∗*^
25	45.2	—/463	300, 255, 151	256, 354	Quercetin-3-O-glucoside
26	47.3	—/609	300	254, 353	Quercetin-3-O-rutinoside
27	48.5	—/447	284, 255, 227	265, 346	Kaempferol-3-O-glucoside
28	55.7	—/301	301, 151	254, 371	Quercetin^*∗*^

**Table 5:** Concentration of predominant compounds (*μ*g/g).

Extract	Quercetin	Caffeic acid	Chlorogenic acid	Gallic acid	(+)-catechin
Black currant pomace	2.18	624.1	517.02	—	314.7
Apricot pomace	—	108.2	704.65	804.4	254.1
Grape pomace	1.87	197.4	604.51	1025	479.7

**Table 6:** The parameters extracted from the cyclic voltammetry curves to characterize the reducing ability of the extracts.

Extract	*I*_p.a_ (*μ*A)	*E*_p.a_ (V)	*E*_p.a_ – *E*_p/2_ (V)	*I*_p,a_/*I*_p,c_
Apricot	2.6	0.54	0.03	—
Black currant	2.3	0.51	0.03	0.96
Grape	3.5	0.48	0.09	2.69

**Table 7:** The reducing power and the radical scavenging activity of the extract.

Extract	Testing technique					
	Reducing power				Antiradical activity	
	Cyclic voltammetry testing		Phosphor-molybdenum method	FRAP reduction of Fe(III)	DPPH radical scavenging assay	ABTS radical scavenging assay
	*I*_p.a_ (*μ*A)	*E*_p.a_ (V)	Milligram of ascorbic acid equivalent/gram extract	Reducing power absorbance at 700 nm	% of radical scavenging activity at 80 mg/mL extract concentration	
Grape	3.5	0.48	571.25	2.84	67.63	55.73
Apricot	2.6	0.54	410.96	2.14	60.58	31.25
Black currant	2.3	0.51	370.45	1.54	41.35	50.00

Each value is mean ± SD (*n* = 3).

**Table 8:** Redox potentials of individual compounds determined with cyclic voltammetry (CV).

Compounds	Oxidation potential (mV)	Anodic peak current (*μ*A)	Reduction potential (mV)	Cathodic peak current (*μ*A)
Caffeic acid	0.43	37	0.27	21
Сhlorogenic acid	0.47; 0.65	75; 122	0.37; 0.21	35; 86
Gallic acid	0.52; 0.79	145; 185	—	—
Quercetin	0.47; 0.62	85; 100	0.38	10
(+)-Catechin (C)	0.37	1.7	0.08	3
L-Ascorbic acid (AA)	0.37	2.8	0.12	1.9
Caffeic acid (CA)+ quercetin (Q)	0.35; 0.58	12.3; 22.6	0.14	10.5
Caffeic acid (CA)+ quercetin (Q)+ L-ascorbic acid (AA)	0.45; 0.71	19; 28	0.12	6
Caffeic acid (CA)+ quercetin (Q)+ L-ascorbic acid (AA)+ сhlorogenic acid (ClA)	0.47; 0.76	19; 33	0.07	6
Caffeic acid (CA)+ quercetin (Q)+ L-ascorbic acid (AA)+ сhlorogenic acid (ClA)+ gallic acid (GA)	0.54; 0.76	16.5; 30	—	—

## Data Availability

All data generated or analyzed during this study are included within the article.
